# Pharmacology of PIEZO1 channels

**DOI:** 10.1111/bph.17351

**Published:** 2024-10-14

**Authors:** Jacob A. Kinsella, Marjolaine Debant, Gregory Parsonage, Lara C. Morley, Muath Bajarwan, Charlotte Revill, Richard Foster, David J. Beech

**Affiliations:** 1 Leeds Institute of Cardiovascular and Metabolic Medicine, School of Medicine, University of Leeds, Leeds, UK; 2 School of Chemistry, University of Leeds, Leeds, UK

**Keywords:** calcium ion, ion channel, mechanical force, non-selective cation channel, pharmacology, pressure, shear stress, small molecule modulator

## Abstract

PIEZO1 is a eukaryotic membrane protein that assembles as trimers to form calcium-permeable, non-selective cation channels with exquisite capabilities for mechanical force sensing and transduction of force into effect in diverse cell types that include blood cells, endothelial cells, epithelial cells, fibroblasts and stem cells and diverse systems that include bone, lymphatics and muscle. The channel has wide-ranging roles and is considered as a target for novel therapeutics in ailments spanning cancers and cardiovascular, dental, gastrointestinal, hepatobiliary, infectious, musculoskeletal, nervous system, ocular, pregnancy, renal, respiratory and urological disorders. The identification of PIEZO1 modulators is in its infancy but useful experimental tools emerged for activating, and to a lesser extent inhibiting, the channels. Elementary structure–activity relationships are known for the Yoda series of small molecule agonists, which show the potential for diverse physicochemical and pharmacological properties. Intriguing effects of Yoda1 include the stimulated removal of excess cerebrospinal fluid. Despite PIEZO1’s broad expression, opportunities are suggested for selective positive or negative modulation without intolerable adverse effects. Here we provide a focused, non-systematic, narrative review of progress with this pharmacology and discuss potential future directions for research in the area.

## Abbreviations

CED: C-terminal extracellular domain
STOML3: Stomatin-like protein 3
GsMTx4: Grammostola Mechanotoxin #4

## 1 Introduction

Eukaryotes as varied as amoebae, moss, flies and humans experience mechanical forces from phenomena such as cell movement, organ structure, tissue contraction, fluid dynamics and gravity. This biology is often tuned to the forces experienced for survival advantage (Fritzsche, 2020). The forces may change with external challenges, disease and tissue stiffening. How forces are sensed and transduced into effects is therefore important to understand. Ion channels often play key roles in force sensing and the PIEZO type of ion channel is pivotal (Coste et al., 2010; Douguet & Honore, 2019; Kefauver et al., 2020). Just two PIEZOs (in humans, **PIEZO1** and **PIEZO2**) confer exquisite force sensing and transduction across diverse membrane, cell and tissue types (Alexander et al., 2023; Coste & Delmas, 2024; Jiang et al., 2021; Murthy et al., 2017; Wu et al., 2017). The PIEZOs assemble as homomeric trimers to form calcium, sodium and potassium-permeable non-selective cationic channels (Jiang et al., 2021). Structural features of the channels have been delineated (Jiang et al., 2021; Yang, Lin, et al., 2022). In the closed state, the channels indent the membrane, forming a bowl-like shape. Above the central ion pore sits a cap-like structure, the C-terminal extracellular domain (CED). Projecting outwards from the pore region are flexible membrane-embedded propeller blade-like features that mediate force sensing. The channels change conformation in response to forces such as increased lateral tension in the membrane, with the ion pore then opening to allow influx of calcium ions (Ca^2+^) and other ions (e.g., the sodium ion, Na^+^), thereby transducing forces into cellular effects. The channels are highly dynamic (Mulhall et al., 2023), responding to force within milliseconds (Coste et al., 2010). They integrate with membrane lipids for ‘force from lipids’ (Cox et al., 2017) and regulated behaviour such as the suppression of inactivation—an adaptation to sustained force (Shi et al., 2020). They interact with other proteins such as MyoD Family Inhibitor Domain Containing to enable altered channel gating (Zhou et al., 2023) and cell adhesion molecules such as cadherins to enable cytoskeletal coupling and cell–cell junction localisation (Chuntharpursat-Bon et al., 2023; Wang, Jiang, et al., 2022). PIEZO1 stands out with its broad, perhaps ubiquitous, expression. It is functional in cells as diverse as adipocytes, cardiac fibroblasts, chondrocytes, collecting duct cells, endothelial cells, enterochromaffin cells, epithelial cells, macrophages, monocytes, myeloid cells, myoblasts, neurones, osteoblasts, pancreatic cells, platelets, red blood cells, smooth muscle cells, stem cells, T cells, tumour cells and urothelial cells (Jiang, Yang, et al., 2021). The numerous functions of PIEZO1 suggested from cell, animal and human studies are not reviewed here but they relate to all major organs and systems (Jiang et al., 2021).

Pharmacology is the science of discovering and investigating drugs and other substances useful in the study of biology and the development of new drugs. Here we focus on the emerging pharmacology of PIEZO1, there being much less progress with the pharmacology of PIEZO2. As yet, there are no therapeutic drugs designed to target the PIEZOs but some drugs designed for other purposes have been found to affect channel function. PIEZO1 pharmacology is therefore largely experimental at present but there is the potential for therapeutic applications, which are being actively explored. Substances that affect PIEZOs can mostly be considered as tool compounds that are helpful for understanding how the channels work, the roles of the channels and the potential of the channels as targets in the treatment of disease. Some of the substances can be classed as small molecule modulators that fit Lipinski rules for therapeutics drugs such as molecular weight of less than 500 (Lipinski et al., 2001). Broad expression of PIEZO1 (Jiang et al., 2021) might argue against its viability as a therapeutic target because multiple unwanted effects might arise. However, specific effects do appear possible, perhaps because of variations in PIEZO1 abundance, lipid associations, mechanical forces and other factors.

## 2 Positive Modulators

### 2.1 Yoda series

#### 2.1.1 Yoda1 discovery

**Figure 1:**
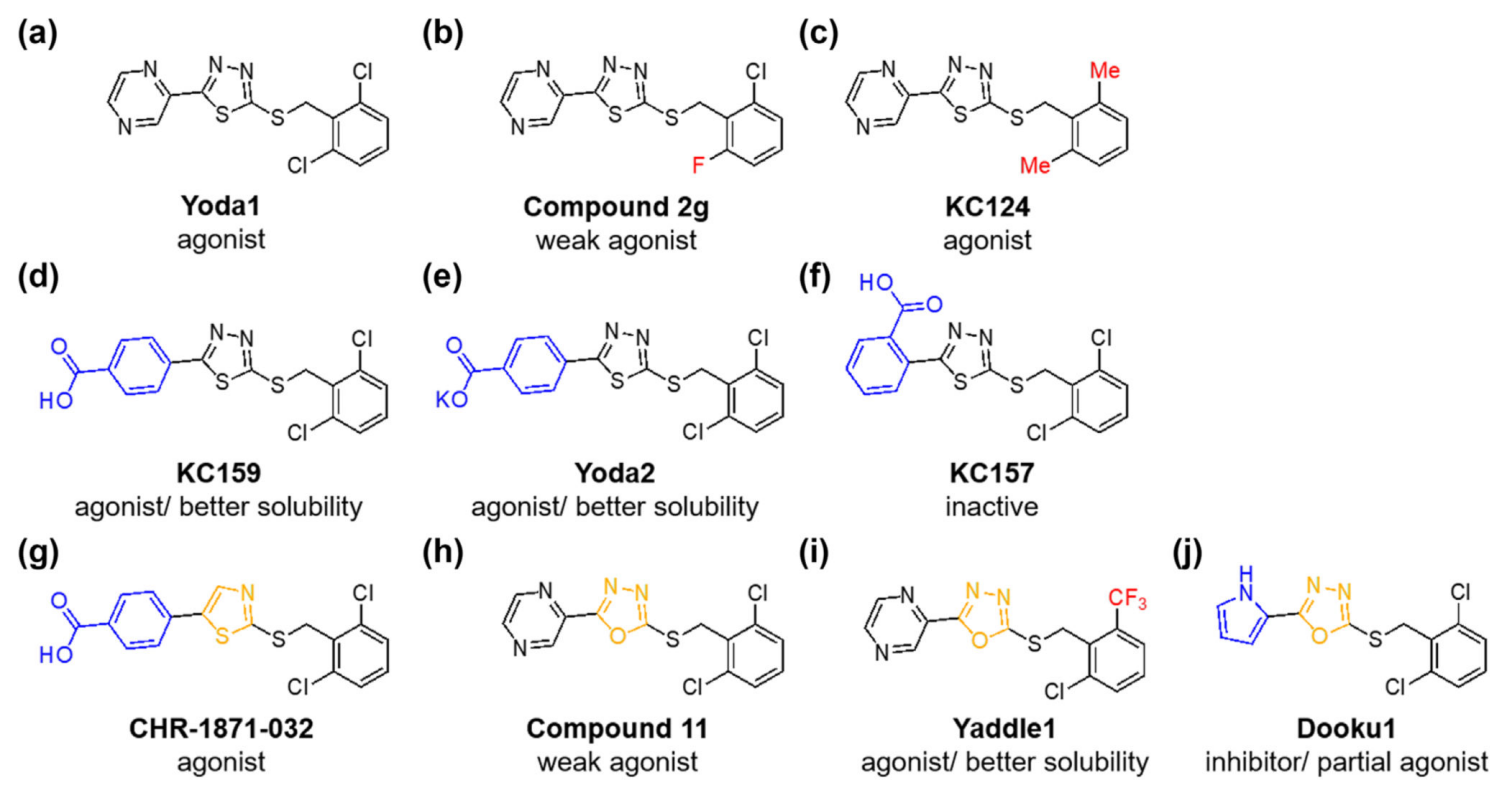
Structures of Yoda series compounds. Chemical differences of analogues compared with Yoda1 are indicated in colour. (a) Yoda1. The 2,6-dichlorophenyl moiety is shown on the right side of the molecule and its pyrazine moiety on the left. The central core is thiadiazole. (b) Compound 2 g. Chemically the same as Yoda1 except for a fluorine (indicated in red) in place of a chlorine in the right-hand ring. Compared with Yoda1, it is a weak agonist of PIEZO1. (c) KC124. Chemically the same as Yoda1 except for methyl groups (indicated in red) in place of the chlorines in the right-hand ring. It has similar or slightly weaker agonist capability at PIEZO1 compared with Yoda1. (d) KC159. Chemically the same as Yoda1 except for a 4-substituted phenyl carboxylic acid (4-benzoic acid group) (indicated in blue) in place of Yoda1’s pyrazine group on the left side. It has similar or stronger agonist capability at PIEZO1 compared with Yoda1. It has better aqueous solubility and other physicochemical properties compared with Yoda1. (e) Yoda2 (KC289). The potassium salt of KC159, also showing improved properties compared with Yoda1. (f) KC157. Chemically the same as Yoda1 except for a two-substituted phenyl carboxylic acid (indicated in blue) in place of Yoda1’s pyrazine group on the left side. It has no or very weak agonist capability at PIEZO1. (g) CHR-1871-032. It is chemically the same as Yoda1 except for a 4-substituted phenyl carboxylic acid (indicated in blue) in place of Yoda1’s pyrazine group on the left side and a monoazole (thiazole) instead of a diazole (thiadiazole) in the central core (indicated in orange). It has slightly stronger agonist capability at PIEZO1 compared with Yoda1 and better capability to rescue loss-of-function variant PIEZO1 channel function. (h) Compound 11. Chemically the same as Yoda1 except for an oxadiazole in the central core (indicated in orange). Compared with Yoda1 it is a slightly less effective agonist at PIEZO1. (i) Yaddle1. Chemically similar to Yoda1 except for the oxadiazole and 2-chloro-6-trifluoromethyl substitution in the phenyl ring. (j) Dooku1. It is chemically the same as Yoda1 except for an oxadiazole in the central core (indicated in orange) and a 2-pyrrolyl instead of pyrazine group on the left. At overexpressed hPIEZO1 channels it lacked agonist activity but it inhibited the action of Yoda1. Additional PIEZO1-related effects of Dooku1 occur (as described in the main text), suggesting that it may have partial agonist capability (i.e., inhibitor or weak agonist capability) depending on context.

About 3.25 million low molecular weight compounds from the Novartis screening collection were tested for their ability to elevate intracellular Ca^2+^ in HEK 293 cells overexpressing mouse PIEZO1 (mPIEZO1) and mouse PIEZO2 (mPIEZO2) (Syeda et al., 2015). About 9,000 compounds caused 50% activation above vehicle control. They were retested in mPIEZO1-, mPIEZO2- and mock-transfected cells, leading to focus on 2-(5-(([2,6-dicholorophenyl]methyl)thio)-1,3,4,-thiadiazol-2-y)pyrazine (Figure 1a) as a PIEZO1 channel agonist. This compound activated mPIEZO1 channels reconstituted in lipid bilayers, suggesting a direct effect on PIEZO1 protein or something closely associated. In patch-clamp experiments, it increased the mechanical sensitivity of the channels, suggesting its classification as a positive modulator. mPIEZO2 was not activated (Syeda et al., 2015) and Ca^2+^ elevation in murine red blood cells was prevented by genetic disruption of PIEZO1 (Cahalan et al., 2015), suggesting PIEZO1 specificity. Concentrations required for 50% effect (EC_50_s) were indicated as 17.1 μM for mPIEZO1 and 26.6 μM for human PIEZO1 (hPIEZO1) but saturating effects were not seen at the maximum concentration used (100 μM) and some of the recording solutions were observed to be opaque, suggesting precipitation of the compound. EC_50_s and physicochemical limitations are discussed below. The compound was named Yoda1, we assume after the catch-phrase ‘may the force be with you’ of the Yoda character in Star Wars films distributed by 20^th^ Century Fox and Walt Disney Studios Motion Pictures. Yoda1 has since been frequently used in this area of research. It and a few analogues are the best pharmacological modulators of PIEZO1 identified so far. The commercial availability of such modulators has positively influenced PIEZO1 research, attracting investigators to the field and enabling the relatively easy manipulation of force sensing pathways, and the testing of roles of PIEZO1 in diverse cell and tissue types, including clinically relevant human samples.

#### 2.1.2 Structure–activity relationships and improvements on Yoda1

The Yoda1 2,6-dichloro substitution of the phenyl ring (Figure 1a) was suggested as being critical for its effectiveness (Syeda et al., 2015). Subsequent research largely supported this perspective, showing data for eight variously substituted phenyl analogues in which seven mostly or completely prevented activity (Evans et al., 2018). A fluorine in place of one of the chlorines (Figure 1b) enabled retention of only weak activity (Evans et al., 2018). Methyl groups in place of the chlorines (Figure 1c) enabled good activity, however (Ludlow et al., 2023). Other activity-retaining alterations of this ring were suggested in a patent application (Li et al., 2021; Tang et al., 2022) and subsequent work showed importance of the position of the chlorine atoms or isosteres of them (Goon et al., 2024). Alterations to the ‘left-hand’ side of the molecule (a pyrazine moiety in Yoda1—Figure 1a) were tolerated and had potential value (Evans et al., 2018; Parsonage et al., 2023). Replacing the pyrazine with a 4-substituted phenyl carboxylic acid (Figure 1d) yielded a PIEZO1 agonist that was at least as good as Yoda1 in potency and efficacy while also improving aqueous solubility and microsomal stability and reducing protein binding (Parsonage et al., 2023). The potassium salt of this analogue (Figure 1e) was named Yoda2 (Parsonage et al., 2023). Specific chemical requirements for channel activation were apparent (e.g., 4-substitution of the carboxylic acid rather than 2- in Yoda2) (Parsonage et al., 2023) (Figure 1d compared with Figure 1f), suggesting the existence of a lock-and-key-like binding site on or close to the channel. Replacement of the central thiadiazole ring was also tolerated, including by similarly substituted thiazole (Figure 1g) (Ludlow et al., 2023) and oxadiazole rings (Figure 1h,i) (Evans et al., 2018; Goon et al., 2024). Combining the oxadiazole with 2-chloro-6-trifluoromethyl substitution in the phenyl ring yielded a compound (Figure 1i) with slightly improved potency compared with Yoda1 and better aqueous solubility (Goon et al., 2024). The compound was named Yaddle1. The name relates to another Star Wars character but ‘Yoda3’ would be more in-keeping with its Yoda1 similarities. Other alterations to the central ring have been explored but they did not enable activity (Goon et al., 2024). Overall the data suggest that new and potentially better PIEZO1 agonists can be achieved based on the Yoda1 template (e.g., Yoda2 [Parsonage et al., 2023] and Yaddle1 [Goon et al., 2024]). The Yoda series contains promising PIEZO1-specific modulators. Their further investigation and development is likely to be worthwhile.

#### 2.1.3 Dooku1

Yoda1 analogues that were weak agonists of PIEZO1 (or apparently inactive) were tested for their ability to inhibit Yoda1-activated PIEZO1 (Evans et al., 2018). Such studies led to interest in 2-([2,-6-dichlorobenzyl]thio)-5-(1H-pyrrol-2-yl)-1,3,4-oxadiazole (Figure 1j), which inhibited the action of Yoda1 but not constitutive channel activity and was named **Dooku1** (Evans et al., 2018). Consistent with a ‘silent binder’ concept (Wijerathne et al., 2022), Dooku1 modulated PIEZO1 single channel behaviour without affecting the channel’s opening probability, contrasting with the effect of Yoda1 (Wijerathne et al., 2022). While Dooku1 inhibited the action of Yoda1 in other studies (Barnett et al., 2023; Deivasikamani et al., 2019; Dela Justina et al., 2023; Kenmochi et al., 2022; Matsunaga et al., 2021; Ogino et al., 2023; Roh et al., 2020; Rong et al., 2024; Wadud et al., 2020; Zeng et al., 2022), intriguing additional effects emerged. Yoda1 promoted calcification of arterial smooth muscle cells and this effect was inhibited by Dooku1 as expected (Evans et al., 2018) but Dooku1 without Yoda1 also inhibited calcification, suggesting a Yoda1-independent effect (Szabo et al., 2022). Dooku1 also inhibited mechanical activation of Ca^2+^ entry in odontoblasts (Matsunaga et al., 2021) and activation of gap junction α-1 protein (**connexin-43, Cx43**) in an osteocyte cell line (Zeng et al., 2022). Topical application of Dooku1 to skin biopsies disrupted dermal-epidermal junctions (Labarrade et al., 2023). Intraperitoneal injection of Dooku1 reduced brain oedema in a mouse model of cerebral haemorrhage (Qu et al., 2023) and Evans blue dye uptake in tibia bone (Zeng et al., 2022). Yoda1 and Dooku1 both caused PIEZO1-dependent enhancement of energy metabolism in endothelial cells, suggesting an agonist effect of Dooku1 (Jiang, Zhang, et al., 2023). Yoda1 antagonist and agonist effects of Dooku1 occurred in red blood cells (Hatem et al., 2023). Dooku1 may therefore be a partial or inverse agonist at PIEZO1, activating or inhibiting the channels depending on context. Actions of exogenous modulators may vary depending on the amount of constitutive channel activity and presence of endogenous modulators such as lipids.

#### 2.1.4 Potency variation and physicochemical properties

Potencies reported for Yoda1 vary substantially. Yoda1 EC_50_s of 0.16–0.23 μM have been determined for activation of Ca^2+^ entry (Evans et al., 2018; Yoneda et al., 2019) and modulation of gene expression occurred at 0.125 μM (Choi et al., 2019), suggesting effects of Yoda1 at nM concentrations and thus much lower than those anticipated from the initial observations and indicated in the above text (Syeda et al., 2015). When considering Yoda1’s potency, it is important to address its aqueous solubility, which is relatively poor in the μM concentration range. The solubility limit of Yoda1 in phosphate buffer is 0.2–3.8 μM (Goon et al., 2024; Parsonage et al., 2023) and so its common use at concentrations of 10 μM or higher in physiological buffers may mean the concentration of Yoda1 that is dissolved and available to the channels is less than indicated. Precipitation out of aqueous solution may depend on assay conditions, which are not standardised between different laboratories or even the same laboratory. An organic solvent may be used to improve the solubility of Yoda1 (such as DMSO) but the amount of it used and the protocol for its use vary. Use of a solvent such as DMSO may affect the softness of cells (Yanamandra et al., 2024) and thereby change the mechanical environment of the PIEZO1 channels and their Yoda1 sensitivity.

There may be other factors that contribute to the absolute potency and, particularly, to explaining effects of Yoda1 in the nM concentration range. Such factors include the duration of exposure to Yoda1 (longer exposure improves its potency (Ludlow et al., 2023)), the host cell or tissue type (e.g., endothelial cells and lymphatics have high sensitivity (Choi et al., 2019; Du et al., 2024; Evans et al., 2018)), native or overexpressed PIEZO1 (e.g., native channels may be more sensitive (Parsonage et al., 2023)), limited accessibility of the channels due to subcellular structures (e.g., cell–cell junctions (Chuntharpursat-Bon et al., 2023)), cell attachment to substrates, mechanical forces in the assay (e.g., due to cell–cell contact) and the type of assay (e.g., fluorescence from an intracellular Ca^2+^ indicator such as fura-2) (Parsonage et al., 2023), a reporter such as FM1–143 (Della Pietra et al., 2023) or genetically engineered Green Fluorescence Protein construct (Yaganoglu et al., 2023), or electrical current detected by patch-clamp (Coste et al., 2010; Parsonage et al., 2023). It is unknown how a factor such as longer exposure to a compound may improve its apparent potency but this could be because there is better equilibration of the compound with the active site, which may allow more complete access of the modulator via a lipid barrier or progress with a forward binding association.

We do not imply criticism of any data set; in our laboratory we observe potency variability in apparently similar conditions. We raise the matter for awareness, particularly for its relevance to studies of structure–activity relationships. In such studies, effects of a novel compound must be carefully compared against a benchmark compound (e.g., a Yoda1 analogue compared with Yoda1). For instance, the kinetic and thermodynamic solubility limits of Yoda2 are 14–27.5 μM in phosphate buffer, at least an order of magnitude better than those of Yoda1 (Parsonage et al., 2023). Therefore, use of Yoda2 as a benchmark and template for new analogues may reduce complications relating to compound solubility. In our experience, better potency, reliability and complete dose–response curves (e.g., curves that reach saturation) are obtained with overexpressed mPIEZO1 rather than overexpressed hPIEZO1. Therefore, using mPIEZO1 as a starting point may be technically helpful when developing new pharmacology for these channels. In general, the investigator needs to be careful about data interpretation and inferences for structure–activity relationships of such compounds. Oil–water partition coefficients are likely to vary for different analogues, affecting their solubility and interaction with the biological site of action, especially if it is membrane-embedded.

### 2.1.5 Enhancement of force-dependent gating

Yoda1 enhances PIEZO1 channel mechanical (pressure) sensitivity, slows channel inactivation (closure during mechanical stimulation), slows channel deactivation (closure after mechanical stimulation) and stabilises the channel open state (Syeda et al., 2015), consistent with the observation that it increases the distance between blades in PIEZO1 channels (Mulhall et al., 2023). It is described as an enhancer of force-dependent gating rather than a force-independent agonist. It can, however, be effective without the experimenter applying exogenous force, perhaps because endogenous forces in cells or between cells and substrates are sufficient to prime the channels. For example, Yoda1 nicely mimics effects of fluid shear stress on endothelium with-out shear stress being applied (Rode et al., 2017; Wang et al., 2016). Given that Yoda1 is a chemical, it lacks the directionality of force and may not activate other force-dependent mechanisms unless they are PIEZO1-dependent, but synergies with actions of forces may lead to force-like effects of such modulators. The potential for synergy between Yoda series compounds and mechanical force is interesting from mechanistic and translational perspectives because it could provide a route to selective modulation (e.g., at sites of fibrosis or focused ultrasound stimuli). Further investigation is needed, however. Is it possible, for example, to predict under what conditions synergy would be strongest? Does synergy need sustained or only transient presence of PIEZO1 modulator? Does synergy occur with modulators other than Yoda series compounds and with other mechanically-sensitive channels?

#### 2.1.6 Evidence for direct interaction

The concept of Yoda1’s direct interaction with PIEZO1 (Syeda et al., 2015) has been supported by results from surface plasmon resonance studies with a purified mPIEZO1 fragment comprising amino acids 1–2190 (Wang et al., 2018) (the full-length protein is 2547 amino acids). The fragment encompassed the majority of the blade region, which is normally mostly membrane-embedded. It lacked the ion pore region, CED or distal C-terminus. The binding dissociation constant was 45.6 μM (Wang et al., 2018), which is 2–22 times higher than EC_50_s of functional effects (Evans et al., 2018; Syeda et al., 2015). The higher value for binding may be explained by considerations such as precipitation of Yoda1 (as discussed above), low mechanical force in the assay (that may reduce channel conformations favourable for interaction), the use of detergent to solubilise the fragment (Wang et al., 2018) (that may remove lipids that improve Yoda1 interaction) or the existence of other, higher affinity, binding sites.

The activating effect of Yoda1 on PIEZO1 channels is seen within 1 s and gradually progresses to a maximum within 10–60 s, when either electrical current or intracellular Ca^2+^ is measured (Lhomme et al., 2019; Rode et al., 2017; Syeda et al., 2015; Wang et al., 2016). Responses may then continue or decay over periods of minutes. Such time courses are not proof for a direct mechanism but do not argue against it either. Methodological considerations and physical factors in the cells complicate measurements of rates and the interpretation of the arising values. Considerations and factors include the speed of application of the substance, the experimental readout and barriers to diffusion such as the lipid bilayer. Mechanical activation of PIEZO1 channels is possible within milliseconds but, in the studies showing this, high-speed pressure pulses or rapid cell indentation (‘poking’) were applied to cells (Wu et al., 2017). Although such speed of delivery is not achieved with Yoda series compounds, small molecule activation does appear to be slower than mechanical activation.

#### 2.1.7 Interaction site and wedge hypothesis

Computer simulations of the central region of mPIEZO1 (including part of the blades) led to the suggestion of a Yoda1 interaction site in a hydrophobic pocket (Botello-Smith et al., 2019). Physical alteration at this site by mutation of alanine (A) to tryptophan (W) at position 2094 inhibited Yoda1 activation of the channels in laboratory experiments (Botello-Smith et al., 2019). Inhibition could have arisen because of reduced binding of Yoda1 at the pocket but other explanations are possible such as conformational changes in the channel that indirectly inhibited the action of Yoda1. The A2094W mutant was suggested to have normal mechanical sensitivity but the mechanical activation curve was altered in shape, the current amplitude was lower and the current kinetics were faster (Botello-Smith et al., 2019). Based on such studies, the idea has been proposed that Yoda1 enhances PIEZO1 force sensitivity by binding at a pocket in the blades, acting like a wedge to increase the susceptibility of the blades to force (Botello-Smith et al., 2019). Further data supporting this idea have come from a cross-linking strategy that reduced Yoda1 effects and computational poses of Yoda1 and Yoda1 analogues in a channel fragment with calculated apparent affinities depending on protein conformation (Jiang, Wijerathne, et al., 2023). The data are consistent with the idea that Yoda1 enhances force-dependent gating.

#### 2.1.8 Selectivity

The original suggestion of Yoda1’s selectivity for PIEZO1 (Syeda et al., 2015) has been supported by results from numerous subsequent studies. Effects of 0.1–50 μM Yoda1 were abolished or strongly reduced by PIEZO1 deletion or depletion in mice or cultured cells (Blythe et al., 2019; Cahalan et al., 2015; Caolo et al., 2020; Choi et al., 2019; Endesh et al., 2023; Lhomme et al., 2019; Li et al., 2019; Liu et al., 2023; Liu, Xu, et al., 2021; Luo et al., 2023; Malko et al., 2023; Morley et al., 2018; Mousawi et al., 2020; Nonomura et al., 2018; Rode et al., 2017; Roh et al., 2020; Suzuki et al., 2018; Swain et al., 2022; Uchida et al., 2021; Wang et al., 2016; Xie et al., 2023; Yang, Zeng, et al., 2022; Ye et al., 2022; Yoneda et al., 2019), suggesting that Yoda1’s many effects on cells, tissues and mice are indeed mediated via PIEZO1. Fewer studies have been performed with Yoda2 but dependence of its effects on PIEZO1 have also been suggested (Parsonage et al., 2023). Selectivity of 5-μM Yoda2 has been investigated in a Eurofins’ Hit Profiling Screen, providing binding data for 30 proteins including ion channels and receptors. It reduced binding of ligands to **adenosine A_2A_ receptors** (40%) and **prostanoid EP_4_receptors** (63%), but it largely lacked effects on binding to the other proteins of the assay (Parsonage et al., 2023). As the EC_50_ values for activation of PIEZO1 by Yoda2 were 0.15– 1.14 μM, the binding data are consistent with Yoda2 having PIEZO1 specificity at suitable concentrations. Endothelial PIEZO1 deletion prevented endothelium-dependent relaxation by 0.1–10 μM Yoda2 (Parsonage et al., 2023). Dooku1 at 10 μM lacked effects on endogenous ATP-evoked Ca^2+^ elevation, store-operated Ca^2+^ entry and overexpressed **TRPV4** and **TRPC4** channels, so it too exhibited PIEZO1 selectivity (Evans et al., 2018).

#### 2.1.9 PIEZO2

Yoda1 or Yoda2 did not evoke Ca^2+^ signals in mPIEZO2-overexpressing cells, suggesting no activation of PIEZO2 (Lacroix et al., 2018;Parsonage et al., 2023 ; Syeda et al., 2015). Consistent with this result, depletion of native PIEZO2 in HeLa cells had only a small effect on Ca^2+^ entry evoked by Yoda1, contrasting with the ~50% reduction caused by PIEZO1 depletion (Parsonage et al., 2023). Similar observations were made for Yoda1 in INS-1832/13 cells (Ye et al., 2022). Yoda2-evoked Ca^2+^ entry in HeLa cells was more significantly reduced after PIEZO2 depletion, however, and depletion of both PIEZO1 and PIEZO2 was more effective against the Yoda2 response than depletion of PIEZO1 or PIEZO2 alone (Parsonage et al., 2023). Yoda2 may therefore be able to activate PIEZO2 channels in some contexts (Parsonage et al., 2023) but further investigation is needed. It could be important to include appropriate exogenous mechanical forces to prime the PIEZO2 channels in the assay. PIEZO2 appears to be more selective in its mechanical sensitivity than PIEZO1 (Jiang et al., 2021), so it may not be primed for chemical activation in routine Ca^2+^ assays, in which exogenous mechanical forces are often not applied.

#### 2.1.10 In vivo use

**Figure 2:**
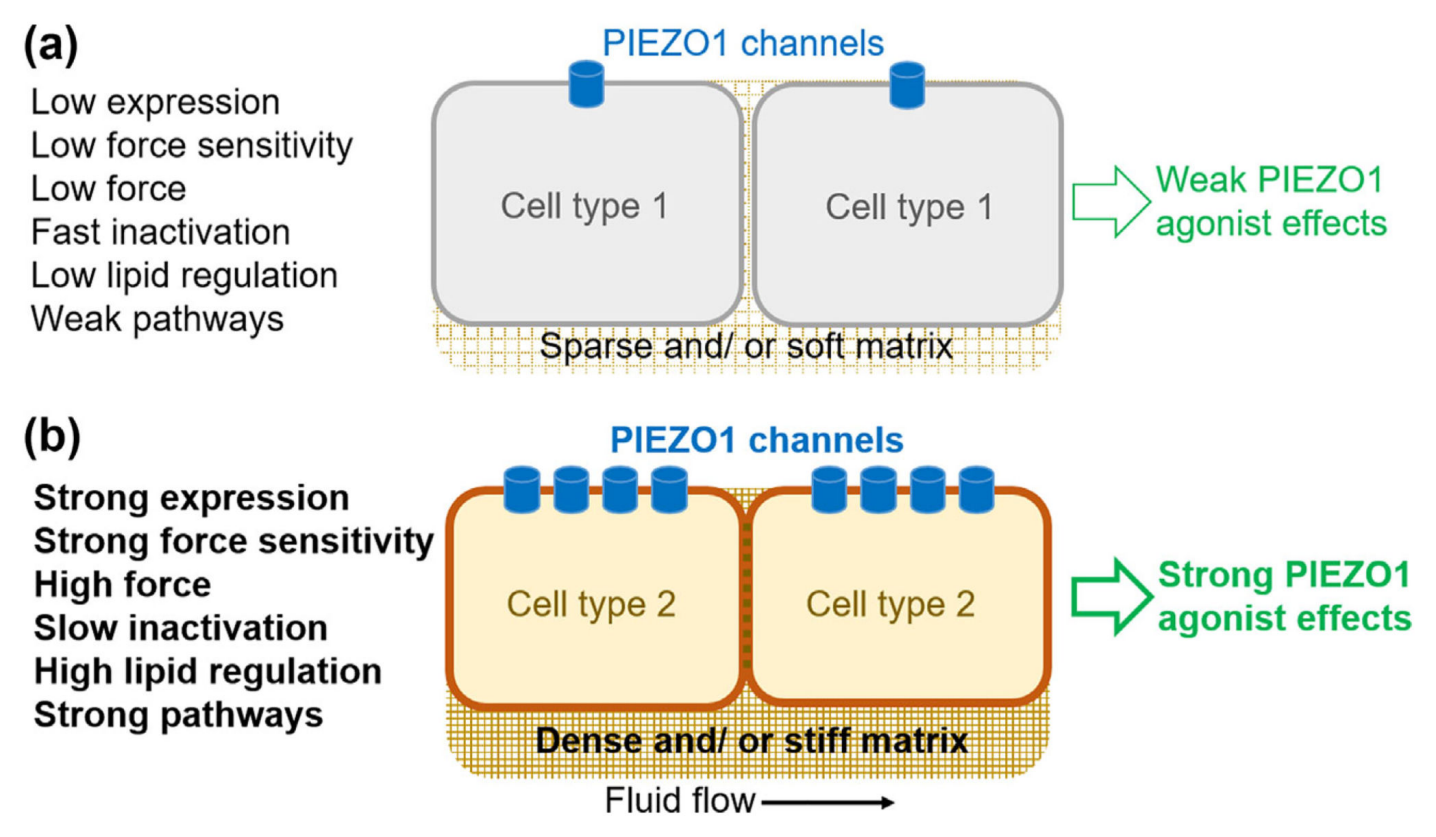
Concepts for cell/ tissue type-specific effects of PIEZO1 agonists. Diagram of contexts predicted to result in weak (a) or strong (b) effects of Yoda series agonists. (a) Organ/ tissue with one or more of: low PIEZO1 channel expression; weak PIEZO1 force sensitivity (e.g., due to loss-of-function PIEZO1 mutation; low force environment (e.g., due to sparse and/or soft extracellular matrix, weak cell–cell contact, little or no shear stress); fast PIEZO1 inactivation (i.e., reducing PIEZO1 channel functional capability); low lipid regulation (e.g., loss of PIEZO1 activity due to depletion of phosphatidylinositol 1, 4, 5-triphosphate; or weak downstream pathways (e.g., due to depleted calpain). An example of Cell type 1 may be physiological cardiac myocytes which, although experiencing a high force context because of the heartbeat, may express very little PIEZO1. (b) Organ/tissue with one or more of: strong PIEZO1 channel expression; strong PIEZO1 force sensitivity (e.g., due to gain-of-function PIEZO1 mutation); strong force environment (e.g., due to dense and/or stiff extracellular matrix, strong cell–cell contact, fluid shear stress); slow or disabled PIEZO1 inactivation (i.e., due to sphingomyelinase activity or MyoD Family Inhibitor Domain Containing protein); high lipid regulation (e.g., due to ω-3 fatty acids); or strong downstream pathways (e.g., due to coupling to nitric oxide synthase). An example of Cell type 2 may be lymphatic endothelial cells.

Despite Yoda1’s unfavourable physicochemical properties for in vivo use (Parsonage et al., 2023), it has often been used in vivo in mice with success, for example by administering repeated intraperitoneal injections (Choi et al., 2024, 2019, 2022; Li et al., 2019; Liu et al., 2023; Matrongolo et al., 2023; Rong et al., 2024; Wang, Yuan, et al., 2022; Xu et al., 2023; Yang, Zeng, et al., 2022; Zhang, Lin, et al., 2023; Zhao et al., 2024; Zhong et al., 2020). Various responses are described, including cardiovascular-related effects, consistent with the prominent expression of PIEZO1 in endothelium (Beech & Kalli, 2019; Li et al., 2014) and the high sensitivity of endothelial PIEZO1 channels to Yoda1 (Choi et al., 2019; Evans et al., 2018). The ability of whole animal systemic Yoda1 to enhance lymphatic structure and function is striking (Choi et al., 2024, 2019, 2022). It is consistent with observations in humans that genetic disruption of PIEZO1 associates with lymphatic disease (Fotiou et al., 2015; Ludlow et al., 2023; Lukacs et al., 2015). Local delivery of Yoda1 has also been explored, including intracranial delivery that improved **β-amyloid** clearance (Jantti et al., 2022), forebrain delivery that reduced memory after sleep deprivation (Zhang, Lu, et al., 2023), nodose ganglion delivery that lowered blood pressure (Cui et al., 2023), intramuscular delivery that reduced muscle atrophy-associated gene expression (Hirata et al., 2022), intragastric deliver that reduced inflammation of intestinal mucosa (Rong et al., 2024) and intravesicular bladder deliv-ery that stimulated urinary voiding (Beca et al., 2021). As described above, Dooku1 has also been used successfully in vivo by intraperitoneal injection (Qu et al., 2023). These Yoda1 and Dooku1 studies have, in most cases, not revealed major adverse effects. Therefore, despite broad expression of PIEZO1 in many cell and tissue types, it may be possible to achieve selective beneficial consequences. This may be because PIEZO1 has different sensitivities to agonists depending on the host cell type and context (e.g., local mechanical forces on cells) (Figure 2a,b). As with most pharmacology, dose and administration are important considerations. Some doses and types of administration may result in adverse effects; for example, local injection of Yoda1 in the tail of rats caused intervertebral disc degeneration (Wu et al., 2022).

#### 2.1.11 Therapeutic drug discovery

The physicochemical properties of Yoda2 are improved over those of Yoda1 (Parsonage et al., 2023) but they are still not optimal. Nevertheless, Yoda2 shows that progress can be made towards a drug-like molecule while retaining and even improving PIEZO1 agonist capability. The chemical structures of Yoda1, Yoda2, Yaddle1 and Dooku1 are, however, published (Evans et al., 2018; Goon et al., 2024; Parsonage et al., 2023; Syeda et al., 2015) (Figure 1). It may be possible to further optimise the Yoda series for commercially viable therapeutic drug development but non-obvious novel PIEZO1 agonists with suitable physicochemical properties and structure–activity relationships may be necessary, or more advantageous. Intracellular Ca^2+^ assays can be used to identify PIEZO1 agonists (Parsonage et al., 2023; Syeda et al., 2015) and so they may be deployed in the future to identify novel activators, ideally in new ways that maximise the chance of revealing relevant new chemical matter. A weakness of Ca^2+^ assays may be that they do not usually incorporate exogenous force, nor make use of the gold-standard recording technique for ion channels, which is patch-clamp; an electrophysiology technique that controls membrane voltage and intracellular and extracellular ionic concentrations, buffers and other constituents. First steps have been made towards high throughput automated planar patch-clamp for PIEZO1 with fluid flow as the stimulus (Murciano et al., 2023). Agonists such as Yoda1 and KC159 (Figure 1d) have been characterised using this approach (Murciano et al., 2023; Parsonage et al., 2023). This patch-clamp innovation offers potential for screening chemical libraries. In addition to developing the chemistry of PIEZO1 modulation, a fruitful approach may be the development of in vivo drug delivery methods for existing modulators (Guan et al., 2023; Yang et al., 2023).

#### 2.1.12 Potential clinical indications and other benefits

**Figure 3:**
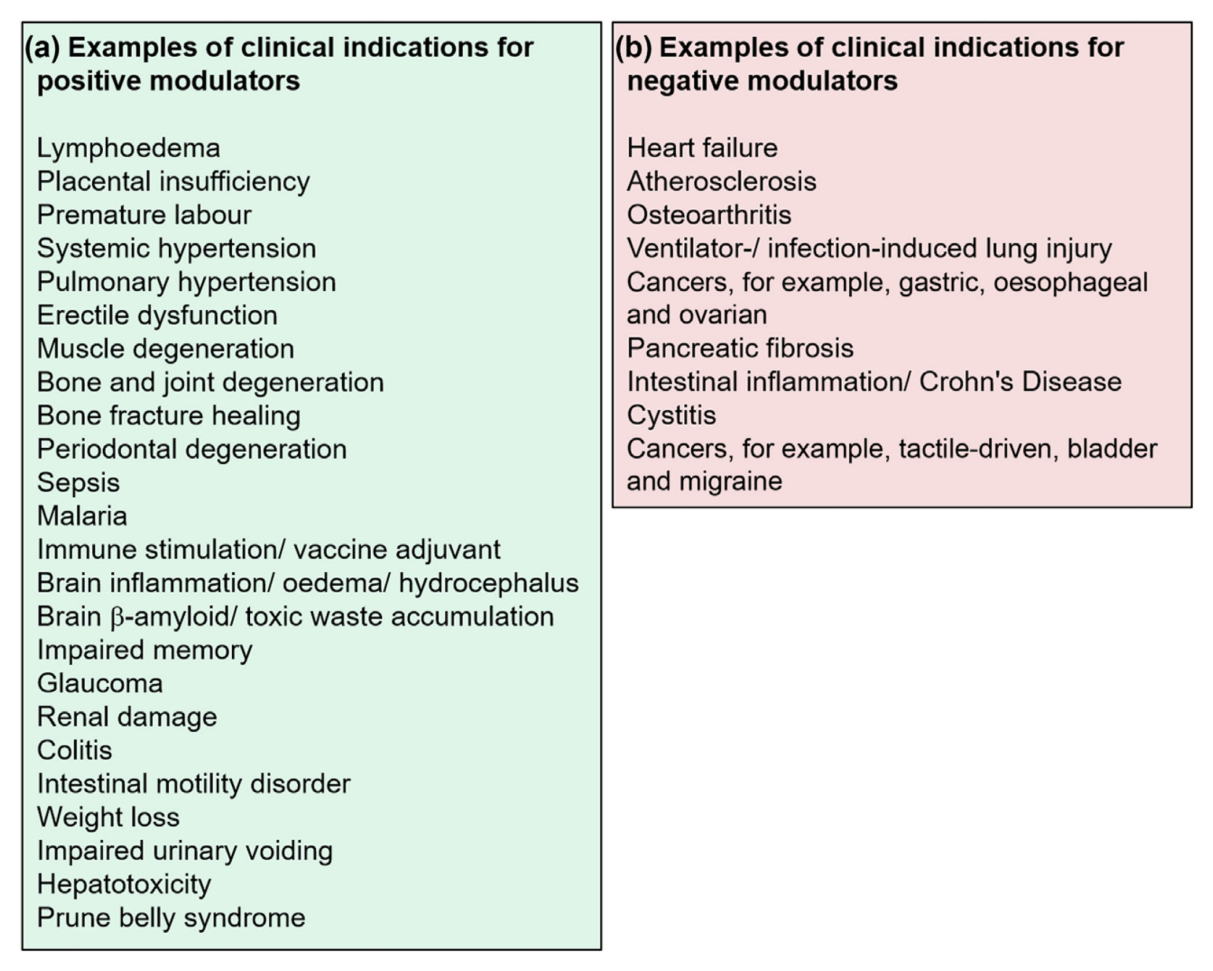
Possible clinical indications for modulators. (a) Positive modulators. The potential uses are suggested based on effects of Yoda series agonists observed in in vivo or ex vivo preclinical experimental studies. (b) Negative modulators. The potential uses are suggested based on effects of PIEZO1 genetic depletion or inhibitors. Potential uses of negative modulators may also be inferred from the suggested contraindications and adverse effects of Yoda series agonists, which are specified in the main text. (a, b) Supporting studies are described and referenced in the main text. The indications are exemplars and not an exhaustive list of what might be possible.

PIEZO1 agonists have been suggested for treating various diseases or their symptoms including lymphedema (Choi et al., 2022; Ludlow et al., 2023), placental insufficiency (Morley et al., 2023), premature labour (Barnett et al., 2023), systemic hypertension (Wang, Yuan, et al., 2022; Yang, Zeng, et al., 2022), pulmonary hypertension (Porto Ribeiro et al., 2022), erectile dysfunction (Dela Justina et al., 2023), muscle, bone and joint degeneration (Bernareggi et al., 2022; Dienes et al., 2023; Guan et al., 2023; Hu et al., 2023; Li et al., 2019), bone fracture healing (Liu et al., 2022), periodontal degeneration (Zhang, Lin, et al., 2023), sepsis (Rong et al., 2024), malaria (Lohia et al., 2023), immune stimulation (vaccine adjuvant) (Goon et al., 2024), inflammation and oedema of the CNS (Malko et al., 2023) including hydrocephalus (Choi et al., 2024), renal damage caused by acute hyperglycaemia (Fei et al., 2023), colitis (Rong et al., 2024), intestinal motility disorder (Xu et al., 2023), weight loss (Zhao et al., 2024), paracetamol-induced hepatotoxicity (Liu et al., 2023), β-amyloid accumulation in the brain (Jantti et al., 2022), toxic waste accumulation in the brain (Matrongolo et al., 2023), glaucoma (Morozumi et al., 2021; Uchida et al., 2021) and prune belly syndrome (Amado et al., 2024) (Figure 3a). Many more possibilities may exist due to the numerous, and varied, functions of PIEZO1 (Jiang et al., 2021); for example, the potential to enhance health benefits of physical exercise has been suggested (Sciancalepore et al., 2022). Although the suggestions of clinical indications and other benefits are based mostly on findings with Yoda1, other chemically distinct PIEZO1 agonists may be similarly useful.

#### 2.1.13 Potential contraindications and adverse effects

The broad expression and many functions of PIEZO1 raise potential concerns about it as a therapeutic target. However, in vivo administration of Yoda1 or Dooku1 in mice seems to be largely beneficial rather than adverse (as described in the above sections). Moreover, gain-of-function PIEZO1 variants are common in some human populations, conferring apparently beneficial rather than adverse consequences (Ma et al., 2018). Furthermore, there is evidence for lymphatic specificity (Choi et al., 2022), suggesting that PIEZO1 agonists (at least at low concentrations) might not uniformly activate PIEZO1 in all cell types. Nevertheless, PIEZO1 research is relatively new and so concerns might arise as we learn more. Adverse effects of PIEZO1 ago-nists have already been observed or might be predicted based on what we know. There is potential for PIEZO1 agonists to cause or exacerbate heart failure (see Beech, 2023), inflammation after myocardial infarction (Sun et al., 2024), vascular calcification (Szabo et al., 2022), lower limb ischaemia (Xie et al., 2023), hypertensive nephropathy (Ogino et al., 2023), thrombosis (Evtugina et al., 2023), cancer (Xiong et al., 2022; Zhang et al., 2022; Zhu et al., 2022), osteoarthritis (Wang, Li, et al., 2022), spinal disc degeneration (Wu et al., 2022), colitis (Leng et al., 2022), anaemia (Cahalan et al., 2015), sickle disease (Nader et al., 2023), pain including tactile-driven and migraine pain (Dolgorukova et al., 2021; Mikhailov et al., 2019; Shin et al., 2023; Wetzel et al., 2017), demyelination and neuronal damage (Velasco-Estevez et al., 2020) and presbyopia (Doki et al., 2023). Indeed, proposals have been made for therapeutic value of PIEZO1 inhibitors (Aykut et al., 2020; Beca et al., 2021; Liu et al., 2018; Pan et al., 2022; Qin et al., 2022; Romac et al., 2018; Shin et al., 2023; Sun et al., 2024; Swain et al., 2022; Wang, Li, et al., 2022; Wetzel et al., 2017; Xiong et al., 2022; Zhang et al., 2018, 2024, 2021) (Figure 3b). However, the strong expression of PIEZO1 in cell types such as endothelium, combined with constant exposure of such cells to mechanical force (e.g., from flow of blood or lymph and pulsatile pressure) and other factors may enable selective amplification of PIEZO1 function by low doses of Yoda series agonists (Figure 2b). PIEZO1 agonists may therefore be a route to new medicines; e.g., that safely enhance lymphatic function and thereby address unmet problems such as lymphedema. The suggestions of contraindications and adverse effects are based on findings with Yoda1 and, in some cases genetic data, but such effects may or may not occur with other types of PIEZO1 activation.

### 2.2 CMPD15

Computational analysis of a chemical library based on knowledge of the Yoda series and its presumed interactions with PIEZO1 identified CMPD15, a potential chemically distinct agonist of mPIEZO1, albeit with lower potency and efficacy than Yoda1 (Jiang, Wijerathne, et al., 2023). Details of the actions and structure–activity relationships of CMPD15 are not yet known. Other novel activators were suggested in the same study (Jiang, Wijerathne, et al., 2023). Computational methods could therefore help to identify novel PIEZO1 agonists and understand their actions, but further development is needed. The approach would ideally be supported by laboratory data showing the structure of PIEZO1 in complex with Yoda1, a Yoda1 analogue or CMPD15. This is, however, challenging to achieve due to the large size of PIEZO1, PIEZO1 dynamics, and the likelihood that the interactions with small molecules are complicated by lipids, which it may not be possible to include in the structural studies or which may be unknown.

### 2.3 Jedi1 and Jedi2

Screening of about 3,000 Tsinghua University and Maybridge chemicals in an intracellular Ca^2+^ assay led to two PIEZO1 activators that may be classed as chemical fragments distinct from Yoda1 (Wang et al., 2018). Chemically related to each other, they are named Jedi1 and Jedi2 (Wang et al., 2018). They activate the channels at high (~mM) concentrations and have binding constants of 2.75 and 2.77 mM at the N-terminal mPIEZO1 fragment (amino acids 1–2190) (Wang et al., 2018). At 0.5–1 mM, Jedi2 mimicked effects of Yoda1 on both red blood cells (Lohia et al., 2023) and principal and intercalated cells of the renal collecting duct (Pyrshev et al., 2023). Progress with this type of agonist is needed if it is to match the capabilities of the Yoda series, ideally with insight into the structure–activity relationships and identification of analogues with potency in the low μM or nM concentration range.

## 3 Negative Modulators

**Figure 4:**
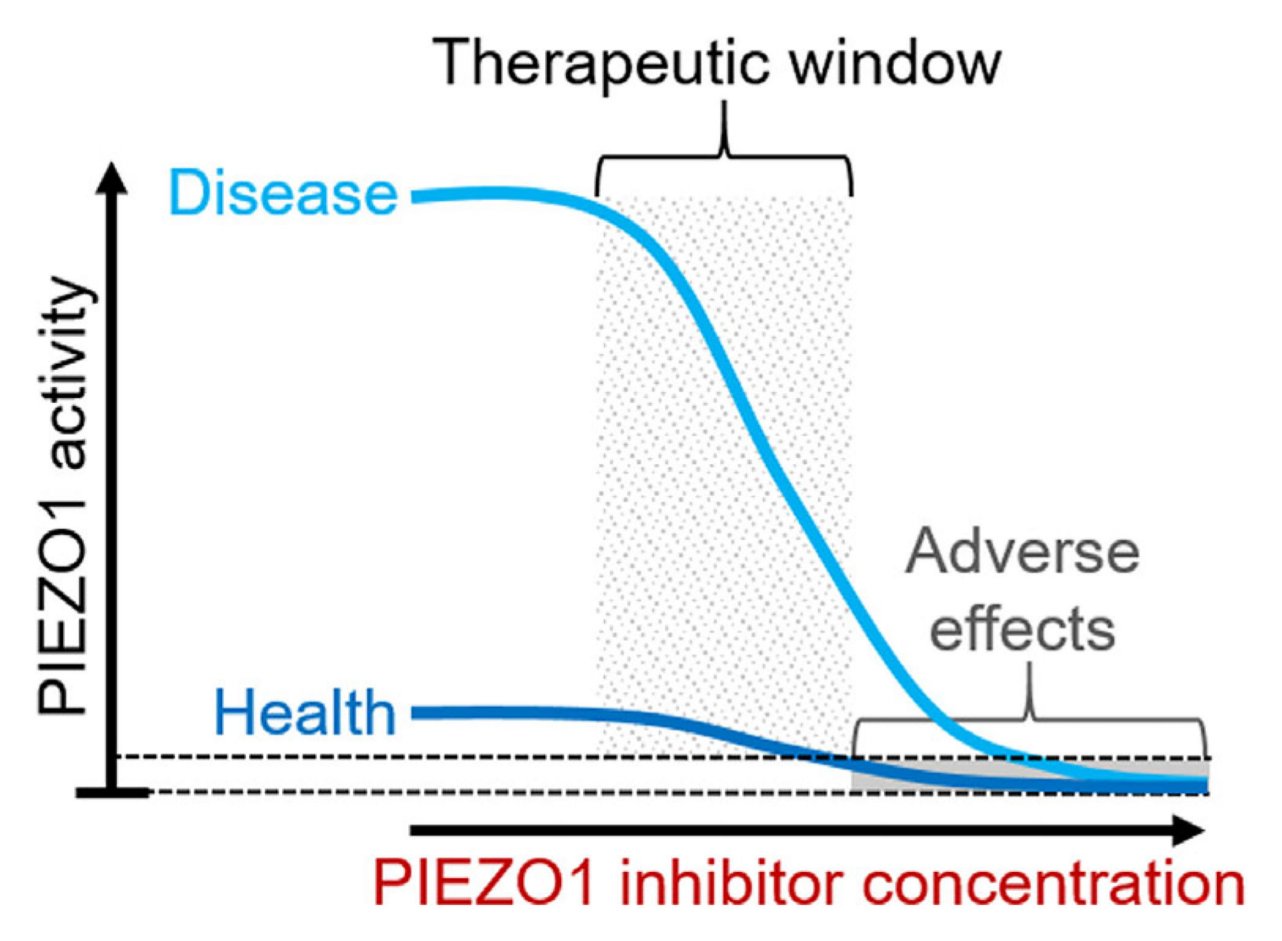
Model of how PIEZO1 inhibitor (negative modulator) therapy might work. The model is adapted from Beech (2023). PIEZO1 expression or activity is assumed to be low in the healthy tissue of the patient but elevated in diseased tissue, in which there may also be increased PIEZO1 expression and/or activity and increased mechanical stress and tissue fibrosis (e.g., in the heart in heart failure). In the model, PIEZO1 inhibitor inhibits the excess PIEZO1, leading to therapeutic benefit. Healthy PIEZO1 may also be suppressed by the inhibitor but adverse (unwanted) effects of the inhibitor (i.e., on healthy tissue) may occur only at supra-therapeutic inhibitor concentrations when there is more than 50% PIEZO1 inhibition. Human genetic studies suggest that 50% loss of PIEZO1 does not have obvious adverse effect. In this model, the partial inhibition of ‘Diseased PIEZO1’ and sparing of some ‘Healthy PIEZO1’ enables a therapeutic window within a specified concentration range of the inhibitor (i.e., depending on dose of the inhibitor and the protocol for its administration). It may, therefore, be possible to achieve therapeutic benefit without unacceptable adverse effects.

Negative modulators that inhibit PIEZO1 function are also sought (Thien et al., 2024). They may have experimental value for determining functions of PIEZO1 and clinical utility as indicated already and outlined in Figure 3b. PIEZO1 is up-regulated in some disease conditions and may have different properties in these settings. PIEZO1 may not operate the same in health and disease if different factors associate with PIEZO1 in the two conditions, so it may be possible to target ‘diseased PIEZO1’. Partly reduced PIEZO1 expression (e.g., due to heterozygous gene variation) occurs without obvious adverse effect in humans (Fotiou et al., 2015), suggesting that partial inhibition of ‘healthy PIEZO1’ by a small molecule might not have unacceptable adverse effect. In this way, a PIEZO1 inhibitor may achieve benefits by reducing adverse effects of ‘diseased PIEZO1’ while sparing enough ‘healthy PIEZO1’ for normal physiological functions (Figure 4).

### 3.1 OB-1 and OB-2

**Figure 5:**
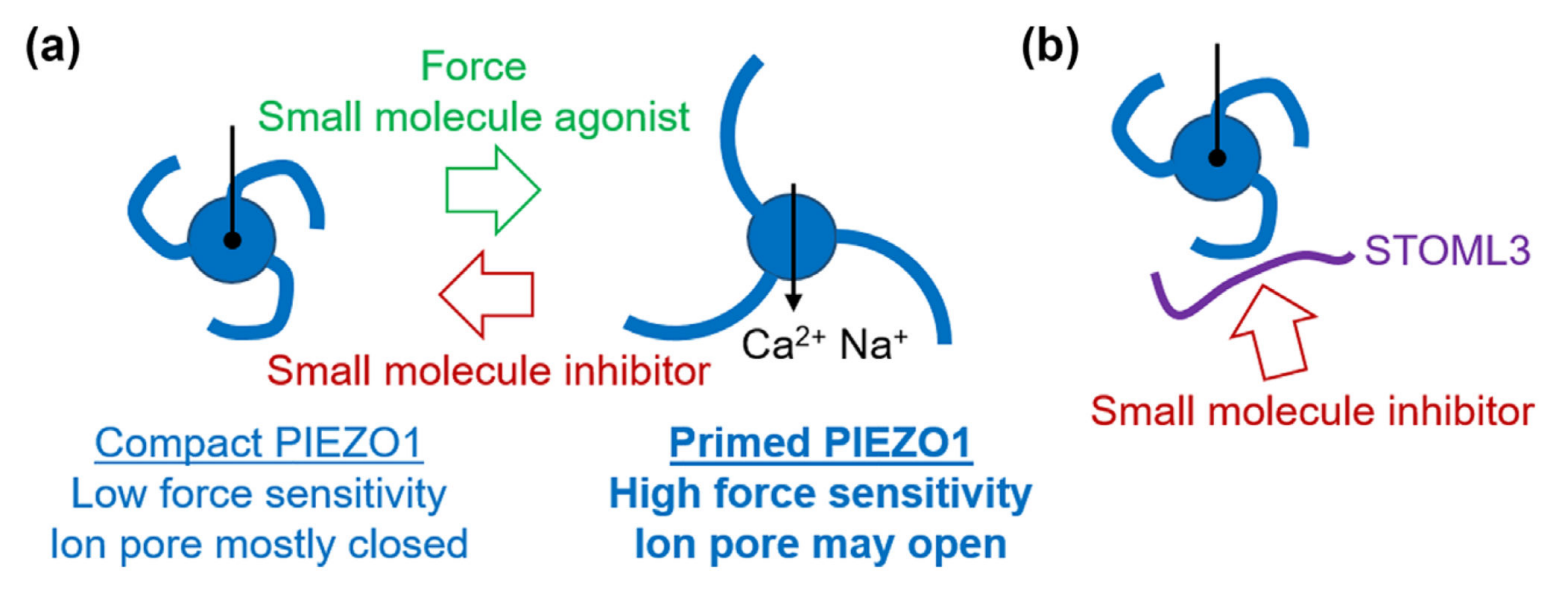
Concepts for PIEZO1 modulation by small molecules. Examples of how small molecule modulation of PIEZO1 may occur. (a) Modulation by Yoda series molecules (or equivalents) that stabilise compact or loose channel conformations that favour ion pore closure (compact conformation) or ion pore opening (loose conformation). PIEZO1 channel (blue) is shown in simple schematic form in helicopter perspective (from above). An agonist such as Yoda1 is suggested to act like a wedge, facilitating activation of the channel by mechanical force. Yoda1 analogues such as Dooku1 may do the reverse: stabilising the compact conformation, yet acting via a similar or overlapping binding pocket. (b) Depiction of the proposed mechanism of PIEZO1 inhibition by OB-1 and OB-2 small molecules, acting via the STOML3 protein. With this mechanism, the inhibition occurs slowly via STOML3 disruption and depends on STOML3 (or a similar molecule) expressed with PIEZO1 (e.g., in tactile neurones).

Stomatin-like protein 3 (STOML3) is an endogenous regulator of PIEZO channels (Poole et al., 2014). Screening of 35,000 small molecules at 10 μM in a STOML3 self-association assay identified molecules OB-1 and OB-2, which, in subsequent assays, were found to slowly (1–3 h) inhibit mechanically activated endogenous PIEZO1 channel currents by 50% at 10 nM (Wetzel et al., 2017). These molecules were therefore suggested to be indirectly acting PIEZO1 modulators, potentially selectively modulating cells containing both STOML3 and PIEZO1 (or PIEZO2) (Wetzel et al., 2017). There are therefore at least two concepts for achieving PIEZO1 inhibition, one of which is a small molecule from the Yoda series (or equivalent) that binds and stabilises a compact channel state (Figure 5a). Dooku1 might act in this way in some circumstances. Another concept is a small molecule that disrupts an associated mechanism such as STOML3 (Figure 5b). OB-1 and OB-2 might act in this way in cell types that co-express STOML3 with PIEZOs (Wetzel et al., 2017).

### 3.2 Benzbromarone

Benzbromarone is a small molecule that has been used in the treatment of gout (Azevedo et al., 2019). It was suggested to inhibit the **Ca^2+^-activated Cl-channel subunit TMEM16A** (Zhang et al., 2013) but this finding has been challenged and instead it has been suggested to inhibit PIEZO1 (Liang et al., 2023). Yoda1-evoked Ca^2+^ entry in red blood cells was inhibited by 50% by 4.3 μM benzbromarone. Further-more, 20 μM benzbromarone almost abolished mechanically-evoked currents in hPIEZO1-overexpressing HEK 293 cells (Liang et al., 2023). Benzbromarone also activated Ca^2+^-activated K^+^ channels, albeit at higher concentrations (Gao et al., 2023).

### 3.3 Inorganic substances

**Gadolinium** (Gd^3+^), in the lanthanide series, has been used as a blocker of various calcium channel types in studies for at least 40 years (Bourne & Trifaro, 1982). Due to similarity in size to Ca^2+^, it can plug channels at or near their ion selectivity filter. Gd^3+^ was used in the original study of mechanically activated mPIEZO1 channels, causing 84% block at 30 μM (Coste et al., 2010). PIEZO2-dependent currents were similarly inhibited (Coste et al., 2010). **Ruthenium red**, a histological dye, has also been used for at least 40 years in research on calcium channels, particularly to block calcium release mechanisms (Miyamoto & Racker, 1982). It blocked PIEZO1 and PIEZO2 channels by about 75% at 30 μM (Coste et al., 2010). However, ruthenium red did not block *Drosophila melanogaster* PIEZO channels, leading to suggestion of a region in mPIEZO1 mediating inhibition by ruthenium red (Coste et al., 2015).

### 3.4 Natural products

#### 3.4.1 Grammostola Mechanotoxin #4 (GsMTx4) and dietary lipids

GsMTx4 is a lysine-rich peptide discovered via a screen of spider venoms against mechanosensitive cationic currents of astrocytes (Suchyna, 2017). At 20 μM, it reduced the inter-blade distance of PIEZO1 channels (Mulhall et al., 2023), consistent with it acting as a PIEZO1 inhibitor (Bae et al., 2011) that promotes a compact (closed) channel conformation (Mulhall et al., 2023). There are effects on channel activity at 10-times lower concentration, with 2.5 μM inhibiting pressure-evoked current through PIEZO1 channels by 71% (D-enantiomer of GsMTx4) or 80% (L-enantiomer) and 4 μM inhibiting cell indentation-evoked currents by 58% (Bae et al., 2011). It has been used quite extensively as a PIEZO1 inhibitor in vitro and in vivo, with beneficial effects suggested such as protection against lung injury during mechanical ventilation (Zhang et al., 2021), bladder hyperactivity (Liu et al., 2018) and neurodegeneration and late-stage demyelinating disease (Velasco-Estevez et al., 2020). It is not selective for PIEZO1, however. GsMTx4 also inhibits currents mediated by PIEZO2 channels (55% inhibition of pressure-evoked currents by 5 μM) (Alcaino et al., 2017), **TRPC5** non-selective cationic channels (98% inhibition hypo-osmotic shock-evoked currents by 5 μM) (Gomis et al., 2008), **TRPC6** non-selective cationic channels (70% inhibition of **uridine triphosphate**-evoked currents by 5 μM) (Spassova et al., 2006) and Na_V_1.1-1.7 sodium channels and **K_V_11.1 and K_V_11.2** potassium channels (50% inhibition of voltage-activated currents by 7.4–14.1 μM) (Redaelli et al., 2010). GsMTx4 may lack specificity because of its mechanism of action which, although still unclear (Mulhall et al., 2023), appears to involve an action on lipids of the membrane, altering lateral membrane tension (Gnanasambandam et al., 2017). Membrane-embedded channels such as PIEZO1 are sensitive to lipid types, and changes in membrane tension caused by different lipids and changes in lipid abundances. PIEZO1 channels are modulated by endogenous lipids such as **phosphatidylinositol 4, 5-bisphosphate** (Borbiro et al., 2015), phosphatidylserine (Tsuchiya et al., 2018) and cholesterol (Chong et al., 2021) and dietary lipids such as cholesterol and the saturated fatty acid margaric acid (Romero et al., 2019) and polyunsaturated fatty acids (e.g., **docosahexaenoic acid**) (Romero et al., 2019). Channels such as PIEZO1 may be particularly sensitive to membrane-modulating agents like GsMTx4 (Cox & Gottlieb, 2019), amyloid β peptides (Maneshi et al., 2018) and lipid changes because PIEZO1 force sensing involves lipid interactions (Cox et al., 2017; Jiang et al., 2021).

#### 3.4.2 Phytochemicals

Screening of 105 plant-derived and synthetic chemicals against Yoda1-evoked Ca^2+^ entry in endothelial cells suggested 9 compounds that inhibit PIEZO1 by at least 75% at 10 μM: salvianolic acid, escin, menthol, trenbolone acetate, 4-hydroxychalcone, cortodoxone (11-deoxycortisol), artemisinin, adrenosterone and jatrorrhizine (Pan et al., 2022). Salvianolic acid B and escin are chemically complex plant-derived substances that have been studied extensively without reference to PIEZOs for therapeutic benefits and anti-oxidant and anti-inflammatory actions (Fazliev et al., 2023; He et al., 2023). Menthol,a simple small molecule, is plant-derived and suggested to act via **TRPM8** channels, causing cold-sensation (Kashio & Tominaga, 2022). Artemesinin has anti-malarial effects and is a prominent plant-derived therapeutic agent (Wells et al., 2015). There is relatively little specific knowledge of 4-hydroxychalcone but many plant-derived chalcones are suggested to be beneficial against cancer and other diseases (Hba et al., 2023). Jatrorrhizine, also plant-derived, is suggested to have various health benefits and mechanisms of action (Rolle et al., 2021). Trenbolone, cortodoxone and adrenosterone are steroids. Further studies of effects on PIEZO1 and PIEZO1-related signals are reported for salvianolic acid B (Grannemann et al., 2023; Pan et al., 2022), escin (Wang et al., 2023), artemisinin (Gan et al., 2023) and jatrorrhizine (Hong et al., 2023). Salvianolic acid B inhibited Yoda1-evoked Ca^2+^ entry in endothelial cells by 50% at 1.37 μM and at 10 μM it inhibited Yoda1-evoked Ca^2+^ entry by about 80% in PIEZO1-overexpressing HEK 293 cells without effect on other Ca^2+^ signals investigated (Pan et al., 2022). Escin inhibited Yoda1-evoked Ca^2+^ entry in endothelial cells by 50% at 1.78 μM (Wang et al., 2023). On a technical note, when escin is used in patch-clamp studies to permeabilize the membrane patch for electrical access (Fan & Palade, 1998), it may inadvertently modulate PIEZO1. Artemisinin (50 μM) partially inhibited Yoda1-evoked Ca^2+^ signals in endothelial and other cell types (Gan et al., 2023). Jatrorrhizine (10 μM) partially inhibited Yoda1-evoked Ca^2+^ signals in endothelial cells (Hong et al., 2023). A separate screen of 92 natural products against Ca^2+^ entry in endothelial cells identified tubeimoside I as a PIEZO1 inhibitor and five other compounds causing at least 70% inhibition of Ca^2+^ entry (Liu et al., 2020). Dose–response analysis suggested that 1.1 μM tubeimoside I inhibited Yoda1-evoked Ca^2+^ entry in endothelial cells by 50% (Liu et al., 2020). The natural products Xueshuantong (Liu, Zhang, et al., 2021) and isoquercitrin (Guo et al., 2024) are also PIEZO1 inhibitors. In summary, diverse phytochemicals (plant-derived chemicals) inhibit PIEZO1 activity in the μM concentration range. No information is yet available on the structure–activity relationships and it may be challenging to determine structure–activity relationships in some cases because of the large and complex nature of the compounds. It is not yet clear if the compounds affect PIEZO1 directly or via intermediates. In contrast to the phytochemicals mentioned above, matrine (a component of herbal medicine) potentiated Yoda1-evoked Ca^2+^ entry after 24-h exposure, suggesting a potential novel route to PIEZO1 enhancement (Jin et al., 2024).

## 4 Discussion and Conclusions

**Figure 6:**
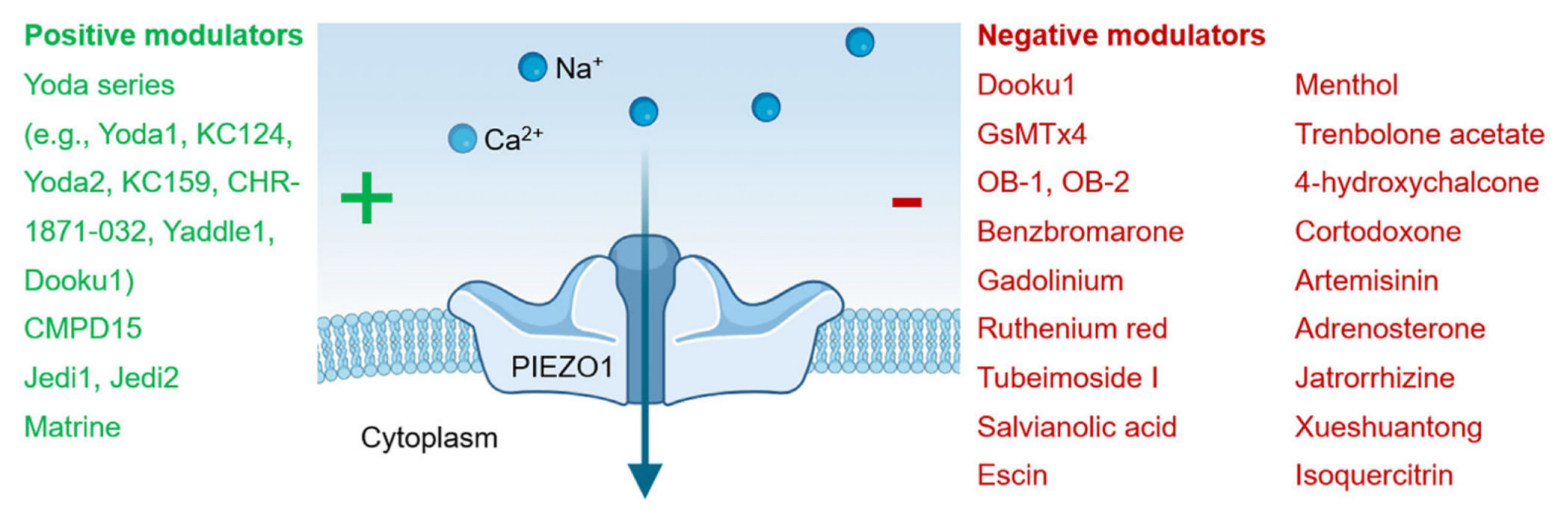
Summary list of PIEZO1 modulators. The blue schematic in the middle is a side-view sketch of the PIEZO1 channel in a lipid membrane, with cations above in the extracellular medium potentially going through the ion pore of the channel once open. The main physiological activator of the channels is mechanical force (e.g., increased membrane tension). Listed on the left in green are substances that have been suggested to activate or enhance PIEZO1 activity by whatever mechanism. Listed on the right in red are substances that have been suggested to inhibit PIEZO1 activity by whatever mechanism. The substances do not necessarily act directly or specifically on PIEZO1. Several independent results and complex data sets are available for some of the substances, whereas for others there may only be one experimental result available. Details of the underlying studies and notes of caution and interpretation are available via the main text. Potential additional modulators have been suggested from results of chemical screens. Other approaches to altering PIEZO1 include RNA interference and gene modification. Anti-PIEZO1 antibody has been used to direct chemical to cells. The side-view sketch of PIEZO1 channel was generated from BioRender.

Since the discovery of mechanically activated PIEZO1 channels (Coste et al., 2010) there has been impressive progress with PIEZO1 pharmacology. Concepts for achieving PIEZO1 modulation are proposed (Figure 5) and there is notable momentum with the emerging class of Yoda series compounds (Figure 1). Yoda1 in particular has been studied by multiple independent research groups and shows promising specificity and apparent direct action on the channels. As yet, other small molecule and natural product modulators remain less understood or studied. Some may not be directly acting or suitable for development but others may provide novel and complementary per-spectives. There are indirectly acting small molecules that may enable context-specific modulation (e.g., OB-1) and the study of effects of existing therapeutic drugs may provide rapid routes to safe inhibition of unwanted PIEZO1 activity (e.g., benzbromarone). The names of positive and negative modulators are summarised (Figure 6). Expression and function of PIEZO1 channels may alternatively be modulated by RNA interference and gene modification (Albuisson et al., 2013; Ludlow et al., 2023). Antibodies targeting the channels may direct chemicals to PIEZO1 (Qin et al., 2022) and could potentially modify channel function themselves (e.g., by promoting internalisation of PIEZO1 protein). Overall, the possibilities are exciting and the idea of PIEZO1 modulation shows tractability and promise. Investigators have a PIEZO1 pharmacological toolkit available (Figure 6) and foundations for developing more modulators and PIEZO1-targeted therapeutics.

The Yoda series contains useful and promising small molecule modulators of PIEZO1, which act mostly as agonists but with potential for inhibition too. Actions in the nM concentration range have been observed and potency data show the potential for improvement through medicinal chemistry. Additional research on the molecules is likely to be beneficial. Such research could expand knowledge of the chemical structure–activity relationships available, determine the specific atomic features required for agonist, partial agonist and antagonist properties and reveal modulators of this type that have better drug-like physicochemical properties and novel chemistry that can be protected for commercial investment. Improvements such as these may be necessary for therapeutically targeting PIEZO1 channels. A modulator binding site has not been definitively identified but there are reasons to think it exists, quite possibly on PIEZO1. Laboratory structural data for the binding site would be helpful to support the idea of the existence of a PIEZO1 chemical-binding pocket and potentially inform the development of improved and novel modulators targeted to such a pocket. It may be possible to obtain such structural insight using advanced cryogenic electronic microscopy or other structural biology approaches in which Yoda1 or analogues such as Yoda2, Yaddle1 or Dooku1 are used. Yoda2 and Yaddle1 might be beneficial because they have improved aqueous solubility and so could be incubated with PIEZO1 protein at higher concentrations than Yoda1 (thereby increasing the probability of observing a small molecule-occupied structural class). Dooku1 may be useful if it stabilises PIEZO1 in a closed conformation, potentially minimising the number of structural classes observed and increasing the resolution of a binding site, ideally at the atomic scale.

In experimental research, it can be helpful to have access to both PIEZO1 activators and inhibitors, but the idea that both might have therapeutic value is challenging. How could it be beneficial and without adverse effects to activate and inhibit the same mechanism? We do not know yet if it could be safe (and effective) but the results of numerous studies suggest that it might be possible. The arguments for developing agonists (rather than inhibitors) are perhaps strongest because we have the Yoda series as a framework and results from animal preclinical studies suggest benefits, as summarised in Figure 3a. The discovery that many people live with a *PIEZO1* gain-of-function variant (Ma et al., 2018) is encouraging from a safety perspective because, while these people are not necessarily unaffected by such variants, major adverse effects seem to be avoided, suggesting that long-term exposure to a PIEZO1 agonist may be tolerable provided that the dose and administration are appropriate. Careful consideration should, of course, be given to potential adverse effects.

The pharmacological toolkit for PIEZO2 is much less advanced than that for PIEZO1. Indirect inhibition via STOML3, ion pore-blocking by Gd^3+^ and potentially indirect inhibition by GsMTx4 are possible, but specific agents and targeted small molecule activators and inhibitors for PIEZO2 are currently lacking. Inhibition of PIEZO2 but not PIEZO1 channels by dioctanoyl phosphatidic acid and palmitoyl lysophosphatidic acid (Gabrielle et al., 2024) might provide starting points for PIEZO2 inhibitor pharmacology. The Yoda series might also provide a route to such pharmacology if there is truly cross-over from PIEZO1 to PIEZO2 (Parsonage et al., 2023). Other routes to PIEZO2 modulation might arise from improved structural resolution (Wang et al., 2019) and screening of chemical libraries for PIEZO2 modulators. Screening may be optimal if it incorporates mechanical activation of the channels in the biological assay, as has been developed for PIEZO1 in automated patch-clamp assays (Murciano et al., 2023).

In conclusion, PIEZO1 pharmacology is available and useful in laboratory and preclinical settings. Further developments of it are likely to be possible and should, in our view, be pursued for improved tools for research. Results from pharmacological studies using such tools provide useful complementary data alongside those of genetic studies, sometimes circumventing limitations of genetic approaches and providing a more effective guide to priorities for drug discovery initiatives. We suggest there are good reasons to explore PIEZO1 activator and inhibitor therapeutics provided that suitable attention is given to key factors such as the clinical indication, pharmacotoxicity, dose and administration route.

### 4.1 Nomenclature of targets and ligands

Key protein targets and ligands in this article are hyperlinked to corresponding entries in http://www.guidetopharmacology.org and are permanently archived in the Concise Guide to PHARMACOLOGY 2023/24 (Alexander, Christopoulos et al., 2023; Alexander, Mathie et al., 2023).

## Data Availability

Not applicable, as this is a review.
